# From anxiety to innovation: the effect of perceived involution on digital creativity via the parallel mediation of relative deprivation and constructive deviance

**DOI:** 10.3389/fpsyg.2026.1822618

**Published:** 2026-05-20

**Authors:** Jinhua Sun, Ziyi Zheng, Jian Hu, Dan Chen

**Affiliations:** School of Management, Chongqing University of Technology, Chongqing, China

**Keywords:** competitive psychological climate, constructive deviance, digital creativity, perceived involution, relative deprivation

## Abstract

**Introduction:**

Against the backdrop of enterprise digital transformation, the increasing displacement of routine roles by technology has intensified workplace competition. This has led to persistently heightened pressure and induced anxiety among employees in a highly competitive environment. Such pressure and anxiety have progressively evolved into a psychological state characterized by diminishing returns cognition and development prospect anxiety, namely, perceived involution, which subsequently affects employees’ willingness to apply and innovate in digital technology.

**Methods:**

This study employed a two-stage questionnaire survey methodology, utilizing a sample of 405 employees from Chinese small and medium-sized enterprises. It constructed a dual-pathway model mediated by constructive deviance and relative deprivation, with the competitive psychological climate serving as a moderator.

**Results:**

The empirical results show that perceived involution exerts a complex dual-path influence on employees’ digital creativity. On the one hand, some employees enhance their creativity through constructive deviance, but on the other hand, more employees experience relative deprivation stemming from this perception, thereby inhibiting creativity. The moderating effect of the competitive psychological climate is asymmetric—it does not amplify the positive pathway but significantly exacerbates the negative pathway.

**Discussion:**

This study reveals the contradictory mechanism of employees’ innovative behavior in an environment of involution and suggests that anxiety and stress play key roles in this process. This provides important management implications for enterprises to optimize their competitive culture from the perspective of mental health care and stress management, alleviate employees’ systemic anxiety, and fully stimulate their innovation potential.

## 1 Introduction

The accelerating pace of digital transformation across global industries has fundamentally reshaped the nature of workplace competition. The World Economic Forum (2016) projected that rapid technological change would render many traditional roles obsolete and place creativity at the center of workforce competitiveness. A subsequent estimate by the McKinsey Global Institute (2017) suggested that as many as 375 million workers worldwide may need to transition to entirely different occupational categories by 2030, driven largely by automation and artificial intelligence. The Gallup State of the Global Workplace Report (2024) documented that employee stress has reached historically elevated levels, with only 23% of the global workforce reporting active engagement. These converging trends indicate that the interplay between technological displacement and psychological strain among employees has become a pressing concern for organizations worldwide.

This phenomenon is particularly salient in East Asian economies, where cultural emphasis on educational attainment and career advancement has long fostered high-intensity competitive environments. In Japan, the deeply rooted overwork culture, or “karoshi” has drawn sustained scholarly and policy attention for decades (Kanai, 2009). South Korea has witnessed a parallel discourse around excessive academic and occupational competition, colloquially termed “Hell Joseon” reflecting youth perceptions of a society with diminishing upward mobility (Cho, 2015). Against this regional backdrop, China has experienced an especially acute manifestation of competitive escalation. As the country undergoes one of the most rapid digital transformations globally, the convergence of technological disruption and labor market saturation has given rise to a distinct cultural phenomenon known as “involution” (neijuan), which has permeated both academic and public discourse since 2020 (Chen B. et al., 2024).

According to data released by China’s Ministry of Industry and Information Technology in 2023, 89% of large-scale industrial enterprises in China have initiated digital transformation projects. However, a survey on digital transformation barriers revealed that 71% of Chinese enterprises identified a talent shortage as the greatest challenge. Some scholars have also noted that the key obstacles to corporate digital transformation often lie not in technological or market changes but in the lack of digital innovation talent to support future strategic development (Ouyang and Zhu, 2025). However, corporate digital transformation demands the participation of all employees. During this process of deep technological integration, the capacity of staff to harness digital tools and unleash new forms of creativity-digital creativity-is pivotal to driving the convergence of digital technology and business operations. A deficiency in digital creativity impedes employees’ ability to master digital tools effectively, hinders their ability to achieve desired performance outcomes through innovative practices such as reconfiguring digital components or developing novel applications for technology, and ultimately obstructs the enterprise’s digital transformation journey (Marsh, 2018). Therefore, enterprises must guide employees in mastering digital technologies while further transforming technical capabilities into higher-order digital creativity to fully unlock the value and potential inherent in digital innovation.

Moreover, the substitution effect of digital technologies on repetitive, routine roles is reshaping career development pathways. This compels individuals to continuously enhance their creativity, particularly their digital creativity and innovation capabilities, with a focus on applying digital technologies to develop sustainable competitive advantages (Lee, 2015).

Simultaneously, the reality of digital technologies gradually replacing routine job functions intensifies workplace competition, leading to a sharply escalating high-intensity competition’ landscape. Consequently, according to shared reality theory, individuals, through social information exchange within the organization, gradually construct a shared cognition of “group competition.” This cognition spreads through interpersonal interaction networks, eventually evolving into an oppressive atmosphere of social consensus, making people feel strong pressure from competition, thus resulting in involutionary behavior (involution). In an environment where involutionary behavior is prevalent, employees gradually develop a systematic understanding encompassing diminishing returns and anxiety about development prospects-known as perceived involution. This psychological perception has permeated the daily work of most digital jobholders and profoundly influences their interaction with digital technologies.

While existing research has explored the impact of leadership behaviors (Öngel et al., 2024), job autonomy (Kaabomeir et al., 2022), and digital work environments (Ferreira et al., 2021) on employees’ digital creativity, the role of individual psychological cognition has been largely overlooked. Particularly in the context of the widespread prevalence of perceived involution, it remains a theoretical black box whether employees, driven by competitive pressure, will stimulate creativity for “innovating to survive,” continuing upwards involution, or, owing to cognitive overload, falling into a “defensive conservatism” pattern, choosing to “lie flat” (tangping). On this basis, this study reveals the mechanism through which perceived involutions influence digital creativity, thereby having significant implications for enterprises undergoing digital transformation.

According to self-determination theory, individuals’ intrinsic psychological needs and autonomous choices play key roles in behavioral motivation. Owing to individual differences and varying organizational climates, employees exhibit differentiated behavioral patterns after perceiving involution. Some employees with stronger autonomy and independence, who prioritize self-feelings, work enjoyment, and a sense of competence and pursue self-actualization, often choose to devote themselves fully to work and strive to stand out in competition when they perceive involution (Zhang and Zhang, 2023). This positive coping style may induce constructive deviance, where employees transcend conventional workflows and rules and take innovative actions to benefit the organization (Luo et al., 2020). Constructive deviance can help employees acquire unique knowledge resources, expand innovation opportunities, and enhance innovation confidence (Galperin and Burke, 2006). When employees have high self-efficacy, they are more confident in trying different methods and approaches, thereby demonstrating better digital creativity. On this basis, this study incorporates constructive deviance as a mediating variable into the research framework to explore the relationship between perceived involution and employee digital creativity.

Upon perceiving involution, others might compare themselves to certain reference standards and perceive themselves as being at a competitive disadvantage, leading to negative feelings of deprivation, i.e., relative deprivation (Yang and Song, 2025). Research by Ye Long’s team at Nanjing University revealed that feelings of psychological deprivation are particularly prominent in explicit comparisons such as performance rankings and promotion opportunities (Ye et al., 2023). For instance, the “rank-and-yank” system implemented by a major internet company directly led to moderate or above anxiety symptoms among some employees, subsequently inducing relative deprivation. Cognition of relative deprivation in the workplace affects employees’ innovative behavior. Research has shown that the relative deprivation felt by Generation Y knowledge workers in the workplace can directly reduce their positive psychological resources, thereby hindering their innovative behavior (Zhou et al., 2024). When innovative behavior occurs in the digital realm, it falls within the core expression of digital creativity. On this basis, this paper introduces relative deprivation as a mediating variable into the framework to discuss the relationship between perceived involution and employee digital creativity.

In highly competitive organizational environments, employees frequently face situations such as performance rankings and competition for promotion opportunities, necessitating social comparison. When individuals believe that “more effort must be invested to maintain a relative advantage,” an involution mindset arises (Li, 2024). Employees transmit pressure signals through defensive competitive behaviors (e.g., ineffective overtime, repetitive labor). The interaction between group imitation and organizational rules solidifies this behavior pattern into collective action, ultimately shaping a strong competitive psychological climate among employees (Li et al., 2022). This competitive psychological climate, in turn, prompts more employees to fall into involution, creating a cycle of “the more involution, the more competition; the more competition, the more involution.” Existing research indicates that when driven by their competitive psychological climate, employees demonstrate stronger work motivation and self-presentation desire, and their work performance often exceeds the average level, which might trigger constructive deviance. Moreover, studies point out that when employees hold a strong competitive psychological climate, those who perceive weak social comparative exchange relationships are prone to experiencing disadvantage when comparing themselves with their colleagues, leading to strong relative deprivation (Tan and Zhang, 2022). Therefore, this study incorporates competitive psychological climate as a moderating variable into the model, deeply exploring its moderating role in the effects of perceived involution on employee digital creativity, as well as the effects of perceived involution on constructive deviance and relative deprivation.

In view of this, this study aims to construct a theoretical model that includes perceived involution, constructive deviance, relative deprivation, and competitive psychological climate. It seeks to explore in depth how perceived involution affects employee’ digital creativity through constructive deviance and relative deprivation, as well as the moderating mechanism of competitive psychological climate in this process. Through this research, we expect to reveal the intrinsic relationship between perceived involution and digital creativity, provide a theoretical basis for organizational managers to formulate effective policies and management strategies, help enterprises foster a healthy competitive environment, stimulate employee’ digital creativity, and achieve sustainable enterprise development in the digital era.

## 2 Hypothesis development

### 2.1 Employees’ perceived involution and digital creativity

Extensive empirical evidence supports the negative link between high-pressure environments and creative output. Byron et al. (2010), in a meta-analysis of 76 experimental studies published in the Journal of Applied Psychology, found that highly evaluative and uncontrollable stressors significantly reduce creative performance by depleting cognitive resources. Anderson et al. (2014), in a state-of-the-science review in the Journal of Management, confirmed that threatening contextual factors within organizations systematically suppress innovative behavior across multiple levels of analysis. Amabile and Pratt (2016), in their updated dynamic componential model of creativity published in Research in Organizational Behavior, further articulated that environments characterized by excessive surveillance, competition, and constrained autonomy erode the intrinsic motivation essential for sustained creative engagement.

Building upon this robust foundation, the current study situates this well-established stressor-creativity dynamic specifically within the context of digital work and the phenomenon of involution. A considerable amount of research has been conducted on creativity. As a major driver of social productivity, it has garnered significant attention from numerous scholars. However, with the progression of the era and the widespread application of digital technology, its influence is now ubiquitous in all aspects of people’s lives, work, and entertainment. It is precisely against this backdrop that digital creativity, a branch of creativity, has begun to attract scholars’ attention. Unlike the traditional concept of creativity, it has been subject to detailed conceptual elaboration. Oldham indicated that digital innovation refers to innovative ideas generated under the influence of digital technology and that the ability to produce and implement these innovative ideas constitutes digital creativity. In other words, the core of digital creativity remains the generation of innovative ideas and their successful implementation. In contrast to traditional creativity, digital creativity is fundamentally based on and driven by the utilization of digital technologies. Notably, the currently prevalent trend of involution within organizational settings undermines this creative process through multiple psychological mechanisms. As competition intensifies in digital work scenarios, Employees’ perceived involution becomes a key factor constraining the release of innovative potential. So-called perceived involution refers to employees’ subjective recognition of “intensified ineffective competition, increased repetitive tasks, and compressed growth space.” Its essence is the individual’s systematic perception of irrational competition within a context of scarce resources (Liu and Qin, 2025).

On the basis of self-determination theory, this study posits that individual intrinsic motivation and autonomy are crucial drivers of creative behavior. An involution environment can diminish employees’ sense of autonomy, causing them to focus their goals narrowly on meeting short-term performance metrics (Chen Y. et al., 2024), rather than pursuing substantive breakthroughs in digital innovation. This deviation in goal orientation makes employees prone to developing a defensive goal orientation, tending to prioritize how to avoid falling behind in intense competition rather than how to create value through digital innovation. When digital technologies are used, employees’ primary consideration often becomes the stability and security of the technology, rather than its innovative potential and possibility for breakthrough; thus, employees exhibit a strong tendency toward patterned use.

Furthermore, from the perspective of conservation of resources (COR) theory, in high-pressure competitive situations, employees, to avoid the risk of resource loss, prioritize allocating cognitive resources to immediate tasks, leading to compressed space for innovative exploration. This defensive resource allocation strategy manifests directly in their preference for using established, standardized digital tools rather than spending time and effort developing novel, customized solutions. Chronic involution pressure can lead employees into states of cognitive rigidity and emotional exhaustion (Zhang and Wang, 2021), severely constraining their use of cognitive resources and making it difficult for them to bear the high cognitive load required for innovative work. Digital innovation often requires employees to engage in open-minded thinking and exploratory behaviors (Huang, 2009), which directly conflict with the state of the resource depletion characteristic of an involution environment.

Additionally, psychological safety, a crucial prerequisite for team learning behavior and innovative performance, is seriously challenged in involution environments. Excessive competition and mutual monitoring in such environments reduce employees’ psychological safety, making them afraid that trial-and-error failures in the digital innovation process negatively impact their performance evaluations and competitive standing. Contextual factors play a key role in creativity. Involution represents a specific organizational context. In this context, when individuals perceive that their psychological resources are continuously diminishing without effective replenishment, protective mechanisms are triggered, reducing innovative exploration to mere technical repetition and trapping them in a vicious cycle of “defensive labor” and superficial innovation. This mechanism is particularly prominent in digital environments because digital innovation typically requires greater tolerance for uncertainty and greater investment in trial and error—precisely what employees in involution environments tend to avoid with their conservative strategies.

The interaction of the aforementioned mechanisms systematically inhibits employees’ creativity in digital environments, inclining them to use standardized, patterned methods to apply technology rather than engaging in impactful digital innovation activities. On the basis of the above analysis, this study proposes the following hypothesis:

*H1:* Employees' perceived involution significantly and negatively influences digital creativity.

### 2.2 The mediating role of constructive deviance

The argument that perceived involution can trigger constructive deviance aligns with the challenge-hindrance stressor framework validated by LePine et al. (2005) in a meta-analysis published in the Academy of Management Journal, which demonstrated that demands appraised as challenges can enhance motivation and performance rather than merely depleting resources. Cavanaugh et al. (2000), in the Journal of Applied Psychology, provided foundational evidence that challenge-related demands are positively associated with job satisfaction and performance, even as they generate strain. Ohly and Fritz (2010), in the Journal of Organizational Behavior, further showed that challenge appraisal of daily work demands is positively linked to proactive behavior, supporting the proposition that involution pressure, when reframed as a challenge, can stimulate unconventional action.

In applying this framework to the involution context, this study explores how such challenge appraisals may manifest as constructive deviance—that is, behavior where employees, despite being knowledgeable about prevailing policies or norms, proactively take breakthrough actions such as optimizing inefficient processes or mobilizing resources across departments to enhance the well-being of the organization or its members (Wang and Hao, 2023). Involution appears to represent a form of urban social pressure stemming from ever-increasing standards and intensifying competition. Conversely, perceived involution focuses on the pressure the subject perceives within the micro-environment (Wang, 2022). It manifests specifically as employees’ subjective experience of scarce workplace resources and intensified competition. In performance-oriented organizations, this experience may prompt some employees, when they perceive involution pressure, to transform it into a challenging stressor, provided that their autonomous motivation is not completely suppressed. Challenge stressors can stimulate employees to think creatively, find solutions, and take innovative actions, thereby inducing constructive deviance (Wang et al., 2019). Such deviant behavior, while seemingly departing from established norms, is ultimately aimed at improving work efficiency and constitutes a positive response to “ineffective involution.”

Furthermore, constructive deviance provides a robust theoretical link to beneficial organizational and innovative outcomes, as evidenced by a strong body of research in high-quality outlets. Vadera et al. (2013), in a comprehensive review published in the Journal of Management, established a theoretical foundation linking constructive deviance to beneficial organizational outcomes, arguing that rule-breaking behaviors oriented toward organizational improvement can generate novel solutions that conventional compliance cannot produce. Galperin (2012), in the International Journal of Human Resource Management, provided empirical evidence that constructive deviance is associated with positive innovation outcomes when employees possess strong organizational identification. Mainemelis (2010), in the Academy of Management Review, developed a theoretical framework demonstrating that creative deviance, defined as the pursuit of novel ideas that violate managerial directives, represents a distinctive pathway to organizational creativity.

This body of work underscores the potential of constructive deviance to serve as a critical mechanism linking perceived pressures to innovative digital behaviors. Specifically, the technology-embedded nature of digital creativity requires employees to use digital technology as the core tool for reconstructing problem-solving paths (Liu and Liu, 2023). However, the inherent rule-based systems within organizations can create path dependence in technology applications. Bureaucratic processes may mandate the use of specific technological tools or adherence to fixed operational standards (Zhang and Zhang, 2023) preventing employees from flexibly integrating emerging digital technologies according to actual needs. In such contexts, organizational constructive deviance—by bypassing redundant approval processes, directly testing new digital technologies, and similar actions—can amend inefficient technology application rules. This shortens the technology validation cycle, accelerates the adaptation of technological tools to specific business scenarios, and thereby unleashes creativity (Zhang and Zhang, 2023).

Secondly, the cross-boundary integration characteristic of digital creativity relies on the flow and recombination of diverse knowledge. However, long-standing “information silos” within organizations can restrict the sharing of data resources. Interpersonal constructive deviance involves breaking down entrenched collaboration practices between departments (Zhang and Zhang, 2023), such as mobilizing data resources across departments without formal permission—reconnecting dispersed knowledge nodes. Furthermore, by stimulating employees’ critical reflection on inefficient rules, perceived involution prompts them to leverage constructive deviance to overcome technological application constraints and knowledge flow barriers. This, in turn, facilitates the enhancement of digital creativity through more flexible and innovative resource integration and technology utilization. Therefore, the following hypothesis is proposed:

*H2:* Employees' perceived involution positively influences constructive deviance.

*H3:* Constructive deviance positively influences digital creativity.

*H4:* Constructive deviance mediates the relationship between Employees' perceived involution and digital creativity.

### 2.3 The mediating role of relative deprivation

The construct of relative deprivation has a well-established theoretical and empirical foundation in social psychology. Smith et al. (2012), in a comprehensive review published in Personality and Social Psychology Review, synthesized decades of research to demonstrate that relative deprivation reliably predicts a range of negative affective and behavioral responses, including resentment, reduced well-being, and withdrawal. Crosby (1976), in a seminal article in Psychological Review, articulated the conditions under which subjective comparisons generate feelings of deprivation, emphasizing that perceived entitlement and feasibility gaps are key antecedents. Pettigrew (2016), in the Annual Review of Psychology, updated the theoretical framework by incorporating both individual-level (egoistic) and group-level (fraternal) deprivation, demonstrating that competitive organizational contexts activate both forms simultaneously.

Anchored in this established theoretical tradition, this study examines relative deprivation as a key psychological mechanism through which perceived involution exerts its negative toll on digital creativity. In highly involuted workplace environments, employees become trapped in excessive competition to achieve the high-load performance targets set by the organization. The resulting persistent stress may induce psychological costs such as job burnout, emotional exhaustion, and generalized anxiety (Liu et al., 2024). The key to understanding this phenomenon lies in relative deprivation theory, which posits that individuals form subjective perceptions of their own workplace status through lateral social comparisons and vertical self-assessments (Zhou and Li, 2011). The involution context further activates this comparison mechanism: at the organizational level, increasing quantitative evaluation standards and the competitive allocation of core resources significantly heighten employees’ sensitivity to relative disadvantage; at the individual psychological level, lateral peer benchmarking and vertical reflection on the “expectation-capability” imbalance constitute the dual core psychological drivers of relative deprivation (Ye et al., 2023).

Relative deprivation refers to the feeling of unfairness or deprivation that individuals or groups subjectively experience after they compare themselves (horizontally or vertically) to a reference group and perceive themselves to be in an inferior position. Its core characteristic is that this feeling stems from the perceived gap resulting from subjective comparison rather than from an absolute lack of objective resources (Guo et al., 2023). According to social comparison theory, when individuals perceive a disadvantage in competition, their psychosocial stress increases significantly, and stress levels form a bidirectional reinforcing relationship with feelings of deprivation. Empirical research supports the dynamic link between workplace relative deprivation and stress responses: employees who perceive their compensation as significantly lower than that of their peers at the same level exhibit a greater tendency toward emotional exhaustion (Zhou et al., 2024). Longitudinal tracking data further confirm that fluctuations in an individual’s sense of deprivation can predict changes in their stress levels (Yang et al., 2015). Building on this, employees’ perception of the involution environment itself, through the stress transmission mechanism, triggers their questioning of the fairness of their own “input-reward” imbalance. This perception of unfairness, in turn, significantly exacerbates the experience of relative deprivation, creating a potentially vicious cycle.

In vertical self-comparison, anxiety arising from the pace of digital skill iteration lagging behind the demands of technological change can induce a sense of deprivation related to “capability-value” imbalance. This perception not only weakens their evaluation of their own digital potential but also reduces their trust in the organization’s technological fairness (Li and Chen, 2022), thereby inhibiting their initiative to explore the innovative boundaries of digital tools. On the other hand, in lateral team comparisons, if individuals experience relative deprivation due to the skewed distribution of digital resources or ambiguous attribution of technical achievements, it may trigger defensive behavioral strategies: either reducing technical collaboration with colleagues to avoid “knowledge spillover” or avoiding high-risk digital trials and errors to maintain a safe competitive zone, ultimately leading to creativity stagnation. In highly involuted workplace environments, employees perceive an imbalance between effort and reward through horizontal and vertical social comparisons, triggering relative deprivation. This deprivation, through anxiety, defensive behaviors, and cognitive resource depletion, weakens employees’ initiative in exploring digital technologies and their breakthrough thinking. On this basis, the following hypotheses are proposed:

*H5:* Employees' perceived involution positively influences relative deprivation.

*H6:* Relative deprivation negatively influences digital creativity.

*H7:* Relative deprivation mediates the relationship between Employees' perceived involution and digital creativity.

### 2.4 The moderating role of competitive psychological climate

Given the profound impact of perceived organizational climate on individual psychological states and behavioral outcomes, this study introduces competitive psychological climate as a key situational factor to explore its moderating effect. Brown and his colleagues (1998), in their analysis of the mechanism through which competitive situations affect sales performance, first defined the core concept of competitive psychological climate, operationalizing it as an individual’s subjective assessment of the strength of the association between organizational resource allocation and horizontal performance comparisons with colleagues. Competitive psychological climate can be understood as an individual-level construct, relating to psychological evaluations of the organizational environment and experiences of competition (Jones et al., 2017). Integrating prior research, this study defines competitive psychological climate as an individual’s psychological perception of “the extent to which competitive behavior is encouraged in the organization,” formed on the basis of their subjective assessment of the strength of the link between organizational resource allocation rules and horizontal performance comparisons with colleagues. In other words, from the employee’s perspective, competitive psychological climate refers to the employee’s perception that organizational rewards, compensation, and promotions depend on comparisons of their performance with that of their colleagues (Brown et al., 1998). In such the competitive psychological climate, employees often experience a strong sense of competition and corresponding work pressure (Beersma et al., 2003; Dong et al., 2024). Research has shown that this perceived climate influences workplace cognition, attitudes, and behaviors (McAllister, 1995; Černe et al., 2013).

On the one hand, when employees perceive the strong competitive psychological climate, the high-pressure and rapidly changing (involuted) team environment increases their tendency toward social comparison. When employees perceive themselves to be in an environment with intensified horizontal comparison, their sensitivity to the effort–reward imbalance is significantly enhanced, thereby accelerating the formation of relative deprivation. On the other hand, high-intensity competitive pressure amplifies the driving effect of perceived involution on constructive deviance; to overcome the impasse of inefficient competition, employees are more inclined to adopt innovative actions that challenge existing organizational rules but benefit overall goals (Zhang and Zhang, 2023). In this case, competitive psychological climate strengthens the conversion path from perceived involution to constructive behavior by enhancing the sense of urgency for change. In summary, this study proposes the following hypotheses:

*H8a:* Competitive psychological climate positively moderates the relationship between employees' perceived involution and constructive deviance. That is, when the competitive psychological climate is stronger, the driving effect of perceived involution on constructive deviance is more significant.

*H8b:* Competitive psychological climate positively moderates the relationship between employees' perceived involution and relative deprivation. That is, when employees perceive a stronger competitive psychological climate, the promoting effect of perceived involution on relative deprivation is stronger.

The intensification of employees’perceived competitive climate exacerbates their perception of resource scarcity and limited development opportunities, thereby significantly enhancing their perceived involution—that is, the intensity of the individual’s experience of ineffective consumption in a state of irrational competition within the organization. When employees perceive a strongly competitive environment, this perception transforms into a challenging stressor (Wang et al., 2019), prompting employees to proactively adopt constructive deviance to break down information silos and hierarchical barriers, accelerating knowledge recombination and cross-boundary integration. Moreover, its ability to challenge the status quo directly stimulates exploratory learning (He, 2018). Together, these processes promote employees‘generation of novel and useful solutions in digital scenarios, ultimately achieving substantial enhancement of digital creativity. Therefore, integrating the relationships revealed by H4 and H8a, this study further proposes a moderated mediation hypothesis:

*H9a:* Competitive psychological climate positively moderates the positive mediating effect of constructive deviance on the relationship between perceived involution and digital creativity. That is, the stronger the competitive psychological climate is, the stronger the positive indirect effect of employees' perceived involution on digital creativity through constructive deviance.

When employees perceived competitive climate continuously intensifies, their sensitivity to the resource distribution structure increases significantly. Employees’ experience of ineffective internal consumption caused by irrational organizational competition (perceived involution) is dramatically amplified. When employees perceive a highly competitive environment, this perception triggers a strong social comparison mechanism (Ye et al., 2023), prompting individuals to frequently compare their compensation, rewards, and development opportunities with those of their colleagues, or leading to a perceived effort–reward imbalance through horizontal self-comparison, thereby fostering relative deprivation. The psychological resource depletion induced by relative deprivation directly compresses the attentional bandwidth available for deep thinking, inhibits the willingness to explore complex digital technologies, causes employees to become conservative in digital application scenarios, and leads to a systematic decrease in digital creativity because of diminished motivation. Therefore, integrating the relationships revealed by H7 and H8b, this study further proposes another moderated mediation hypothesis:

*H9b:* Competitive psychological climate positively moderates the negative mediating effect of relative deprivation on the relationship between perceived involution and digital creativity. That is, the stronger the competitive psychological climate is, the stronger the negative indirect effect of employees' perceived involution on digital creativity through relative deprivation.

**Figure 1:**
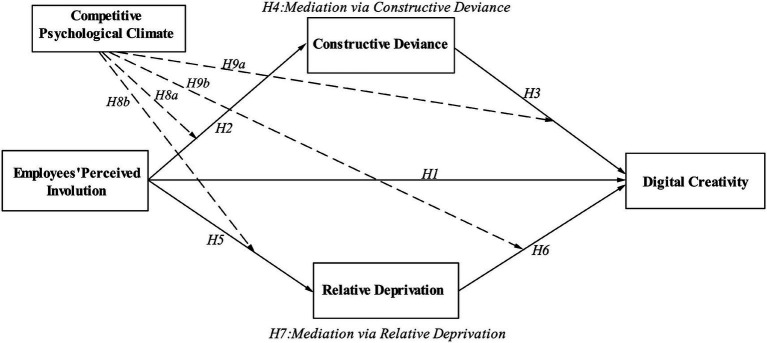
Theoretical model.

On the basis of the above analysis and theoretical reasoning, the theoretical model of this study is shown in Figure 1.

## 3 Research method

### 3.1 Research procedure and sample

In this study, employees from small and medium-sized enterprises undergoing digital transformation in provinces and cities such as Chongqing, Sichuan, and Shanxi were selected, primarily involving industries such as manufacturing and internet-related sectors. Data collection was conducted mainly through field surveys and online platform questionnaires. Through these methods, the researchers contacted the leaders of 70 work teams. The researchers subsequently explained the purpose and procedure of the study to these team leaders and guaranteed the confidentiality of the survey. After providing written informed consent, they were asked to provide a list of all direct subordinates on their teams. The researchers then randomly selected several subordinates from these lists as matching subjects and requested that the team leaders provide contact information for these subordinates. With respect to the subordinate participants, the researchers also explained the survey process and precautions, emphasizing the importance of providing truthful responses. The formal survey proceeded only after their written informed consent was obtained. To reduce common method bias, this study employed a two-stage time-lagged approach to collect leader-employee matched data, with a three-week interval between the two survey waves.

This study adopted a multi-stage cluster sampling approach combined with stratified purposive selection. The research team identified 70 work teams across SMEs in Chongqing, Sichuan, and Shanxi that had implemented digital transformation initiatives and employed digital technologies in daily operations. Within each participating team, a random selection of subordinate employee was conducted to construct leader-employee dyads. The minimum required sample size was determined using the formula proposed by Cochran (1977), where *n* = Z^2^pq/e^2^, with a 95% confidence level (*Z* = 1.96), an assumed proportion of *p* = 0.5 to maximize sample size, and a margin of error of e = 0.05, yielding a minimum required sample of 385. The final valid sample of 405 matched dyads exceeded this threshold. This sampling strategy was chosen for three reasons. Multi-stage cluster sampling is well suited to organizational research contexts where complete employee rosters are available only at the team level (Hox et al., 2017). Stratified purposive selection of enterprises ensured adequate representation of both manufacturing and internet-related sectors. Random selection within teams mitigated self-selection bias and enhanced the representativeness of within-team variation.

In the first stage, employees were asked to complete questionnaires measuring their perceived involution, demographic characteristics, occupational type, and information regarding their exposure to digital technologies such as big data. A total of 622 questionnaires were distributed. After questionnaires were excluded from respondents who reported no exposure to digital technologies, those with clearly patterned responses, and those with excessive missing items, 502 valid questionnaires were retained. In the second stage, the same respondents who provided valid first-stage questionnaires and their direct supervisors were targeted for the second survey, which measured perceived involution, competitive psychological climate, relative deprivation, constructive deviance, and digital creativity. Digital creativity was rated by the employees’ direct supervisors; each leader evaluated 3 to 6 of their subordinate employees. The remaining variables were self-reported by employees. A total of 114 leader questionnaires and 502 employee questionnaires were distributed. After matching and deleting invalid responses, 405 valid matched questionnaires were ultimately obtained, resulting in a valid response rate of 79.7%.

### 3.2 Measurement tools

In this study, maturity scales were used to measure the five key variables: perceived involution, relative deprivation, constructive deviance, competitive psychological climate, and digital creativity. All the scales utilized a five-point Likert scale.

#### 3.2.1 Perceived involution

This study primarily referenced the scale developed by Chinese scholar Yang (2022). The scale consists of 12 items across four dimensions: subsistence need perception (3 items), self-improvement perception (3 items), incentive promotion perception (3 items), and group comparison perception (3 items). A sample item is “To maintain my livelihood, I need to put in significant effort.” The Cronbach’s *α* coefficient for this scale was 0.966, indicating good reliability.

#### 3.2.2 Relative deprivation

This study employed the scale developed by Tropp and White (1999), which includes 3 items covering cognitive and affective dimensions. A sample item is “In my job, I actually receive less than I deserve.” The Cronbach’s α coefficient for this scale was 0.835, indicating good reliability.

#### 3.2.3 Constructive deviance

This study used the scale compiled by Wang and Cui (2018). This scale, developed on the basis of the Chinese context, consists of 7 items across two dimensions: violating formal norms and violating informal norms. A sample item is “I might not comply with company rules and regulations to help other colleagues.” The Cronbach’s α coefficient for this scale was 0.943, indicating good reliability.

#### 3.2.4 Competitive psychological climate

This study used the 4-item scale developed by Brown et al. (1998). A sample item is “My supervisor frequently compares my performance with that of my colleagues.” The Cronbach’s α coefficient for this scale was 0.869, indicating good reliability.

#### 3.2.5 Digital creativity

This study adopted the three-item scale developed by Li et al. (2022) to measure employees’ digital creativity, with all items rated by direct supervisors. The three items, respectively, capture three core facets of digital creativity as conceptualized by Lee (2003) and elaborated by Lee and Chen (2015). The facet of digital knowledge creation assesses whether employees generate new knowledge through digital technology use. The facet of digital problem-solving assesses whether employees devise novel solutions to work challenges by leveraging digital tools. The facet of digital idea implementation assesses whether employees successfully translate digitally-enabled ideas into practical work outcomes. Together, these three facets encompass the full cycle of digital creativity, from ideation through knowledge generation to practical application, consistent with the theoretical definition that digital creativity involves all forms of creativity driven and facilitated by digital technologies. A sample item (rated by supervisors) is “By using digital technology, this employee can continuously create new knowledge in their work.” This parsimonious three-item structure has been widely adopted in studies of digital creativity in organizational contexts. Ouyang and Zhu (2025), in a study published in Soft Science, employed the same three-item scale to examine the impact of workplace digitalization on employee creativity. Kang and Yoo (2025), in a systematic review of digital creativity in the workplace published in Human Resource Development Review, identified this three-item measurement approach as one of the established operationalizations of digital creativity at the individual level. The Cronbach’s *α* coefficient for this scale was 0.906, and the CFA results (Table 1) confirmed that the five-factor model including this scale demonstrated adequate fit (RMSEA = 0.062, CFI = 0.929), indicating satisfactory reliability and construct validity.

**Table 1:** Results of the confirmatory factor analysis.

Model	Χ2	df	Χ2/df	RMSEA	CFI	TLI	SRMR
Five-factor model	813.378	367	2.216	0.062	0.907	0.922	0.047
Four-factor model	1634.232	371	4.405	0.103	0.800	0.781	0.159
Three-factor model	3532.724	374	9.446	0.163	0.500	0.457	0.242
Two-factor model	3606.774	376	9.592	0.164	0.489	0.448	0.246
Single-factor model	4099.548	377	10.874	0.176	0.411	0.365	0.249

*n* = 405. All Δχ^2^ values are significant at *p* < 0.01.

To examine whether the nested structure of employees within teams would necessitate multilevel modeling, we calculated the intraclass correlation coefficient (ICC(1)) for the dependent variable—employee digital creativity. The ICC(1) value was 0.01, indicating that only 1% of the total variance in digital creativity resided between teams, while 99% resided within teams (i.e., at the individual level). According to conventional guidelines (ICC < 0.05 suggests negligible clustering effect; Bliese, 2000), this result implies that the data do not exhibit significant non-independence due to team membership. Therefore, multilevel modeling is not required, and single-level path analysis (as employed in this study) is statistically appropriate.

## 4 Data analysis and results

### 4.1 Confirmatory factor analysis

All data analyses were conducted using Mplus 8.3 (Muthén and Muthén, 2017). Mplus was selected as the primary analytical tool for several reasons. It provides robust maximum likelihood estimation with standard errors that are robust to non-normality (MLR estimator), which is appropriate given the ordinal nature of Likert-scale data (Finney and DiStefano, 2013). Mplus offers an integrated framework for conducting confirmatory factor analysis, structural equation modeling, mediation testing via bootstrapped confidence intervals, and latent interaction modeling within a single analytical environment, thereby avoiding the inconsistencies that may arise from transferring data across multiple software platforms. Its capacity to simultaneously model complex mediation and moderation effects, including the computation of conditional indirect effects at different levels of the moderator variable, made it particularly suitable for testing the moderated mediation hypotheses (H9a and H9b) in the present study. Specifically, all models were estimated in Mplus 8.3 using the MLR estimator (maximum likelihood with robust standard errors). Indirect and conditional indirect effects were tested with bias-corrected bootstrap confidence intervals based on 5,000 resamples, implemented via the MODEL INDIRECT command with VIA statements for the two parallel mediators. Latent interaction terms for H9a and H9b were constructed using the XWITH command under TYPE = RANDOM with numerical integration. To enable independent verification, the complete Mplus syntax and the relevant abridged output (parameter estimates, standard errors, and bootstrap confidence intervals for all mediation and moderated-mediation paths) are provided in the online Supplementary Appendix A. In this study, Mplus 8.3 was used to perform confirmatory factor analysis (CFA) on the five research variables. As shown in Table 1, the results of the CFA indicated that the hypothesized five-factor model demonstrated a significantly better fit to the data than the other four alternative models did (RMSEA = 0.062, CFI = 0.929, TLI = 0.922, SRM*R* = 0.047). These results suggest that the five variables in the research model possess good discriminant validity.

Prior to conducting structural equation modeling (SEM) analysis, it is necessary to examine the distributional characteristics of each latent variable to satisfy the underlying assumptions of model estimation. This study employed skewness and kurtosis indices to test the normality of the variables, adopting the general SEM guidelines proposed by Byrne (2016): absolute skewness values less than 2 and absolute kurtosis values less than 7 indicate that the variables meet the requirements for approximate normality and are suitable for maximum likelihood (ML) estimation. As shown in Table 2, the absolute skewness values for the five core variables in this study range from 0.037 to 0.217, which are substantially below the stringent threshold of 2. None of the variables exhibit extreme skewness, indicating favorable distributional symmetry. The absolute kurtosis values range from 1.094 to 1.472, also well below the stringent threshold of 7, with no evidence of extreme kurtosis.

**Table 2:** Results of normality test.

Variable	Skewness	Kurtosis
Employees’ perceived involution	0.204	−1.472
Constructive deviance	0.166	−1.440
Relative deprivation	0.128	−1.129
Competitive psychological climate	0.037	−1.094
Digital creativity	−0.217	−1.214

In summary, all core variables in this study satisfy the requirement of approximate normality and present no serious non-normality issues. Consequently, maximum likelihood estimation can be appropriately employed for subsequent structural equation modeling analysis, thereby ensuring the validity and robustness of the model parameter estimates.

### 4.2 Common method bias

To assess the potential influence of common method bias, we first conducted Harman’s single-factor test. The unrotated exploratory factor analysis showed that the first factor accounted for 32.66% of the total variance, below the conventional threshold of 40%. However, this heuristic test has limited diagnostic power. A more rigorous defense against common method bias rests on several features of our research design and empirical evidence. First, we employed a two-wave time-lagged design with a three-week interval between measurements, which reduces the risk that responses are unduly influenced by transient mood states or immediate recall. Second, the dependent variable “digital creativity” was rated by direct supervisors rather than by employees themselves, thereby eliminating same source inflation for the key outcome. Third, the confirmatory factor analysis results presented in Table 1 demonstrate that the hypothesized five-factor model fits the data significantly better than any alternative, more parsimonious models (e.g., four-factor, three-factor, and single-factor models). This pattern of discriminant validity is unlikely to arise solely from common method variance (Podsakoff et al., 2003). Taken together, these design and statistical features suggest that common method bias does not seriously distort the findings of this study.

### 4.3 Descriptive statistics and correlation analysis

As shown in Table 3, Employees’ perceived involution was positively correlated with constructive deviance (*r* = 0.378, *p* < 0.01), suggesting that employees who perceived higher levels of involution were more likely to exhibit constructive deviance. This may indicate that in an involuted environment, some employees attempt to cope with pressure through innovative means. Employees’ perceived involution was strongly positively correlated with relative deprivation (*r* = 0.562, *p* < 0.01), implying that employees with stronger perceived involution were more prone feeling disadvantaged in terms of resources and rewards than others were. As these are zero-order correlations, they are consistent with but do not, on their own, establish a directional or causal relationship between the variables; causal interpretations are deferred to the structural model. Employees’ perceived involution was positively correlated with competitive psychological climate (*r* = 0.382, *p* < 0.01), indicating a synergistic relationship between perceived involution and organizational competitive psychological climate. A high-competition environment may intensify employees’ experience of involution, and vice versa. Employees’ perceived involution was negatively correlated with employee digital creativity (*r* = −0.315, *p* < 0.01), suggesting that stronger perceived involution was associated with lower digital creativity, possibly because the pressure or rigid work patterns induced by involution inhibited innovative willingness.

**Table 3:** Correlation analysis.

Variable	1	2	3	4	5
Employees’ perceived involution	1				
Constructive deviance	0.378**	1			
Relative deprivation	0.562**	0. 174**	1		
Competitive psychological climate	0.382**	0. 170**	0.484**	1	
Employee digital creativity	−0.315**	0.203**	−0.314**	−0.169**	1

*n* = 405. **p < 0.01 (two-tailed).

Constructive deviance was weakly positively correlated with relative deprivation (*r* = 0.174, *p* < 0.01), indicating that employees with greater relative deprivation might attempt to break norms because of dissatisfaction with the status quo, albeit the relationship is weak. Constructive deviance was positively correlated with competitive psychological climate (*r* = 0.170, *p* < 0.01), suggesting that the competitive psychological climate might encourage employees to engage in constructive deviance to gain an advantage and that both may be driven by organizational culture. Constructive deviance was positively correlated with employee digital creativity (*r* = 0.203, *p* < 0.01), suggesting a potential synergy between constructive deviance and digital creativity, as employees who proactively break conventions are more inclined to experiment with digital technology innovation.

Relative deprivation was strongly positively correlated with competitive psychological climate (*r* = 0.484, *p* < 0.01), indicating that a more intense competitive psychological climate was associated with stronger feelings of relative deprivation among employees. Relative deprivation was negatively correlated with employee digital creativity (*r* = −0.314, *p* < 0.01), suggesting that employees who are dissatisfied with resource allocation may lack the motivation to engage in digital innovation or that their negative emotions may inhibit creativity.

A negative correlation was found between competitive psychological climate and employee digital creativity (*r* = −0.169, *p* < 0.01), indicating that more intense competition is associated with lower levels of digital creativity among employees. This may be related to the short-term utilitarian focus, reduced cooperation, or work overload resulting from high competition.

### 4.4 Hypothesis testing

#### 4.4.1 Direct effects testing

As shown in Table 4, after controlling for all the demographic variables, employees’ perceived involution had a significant negative effect on employee digital creativity (*β* = −0.371, *p* < 0.001). Therefore, H1 was supported. Employees’ perceived involution had a significant positive effect on constructive deviance (*β* = 0.366, *p* < 0.001). Therefore, H2 was supported. Constructive deviance had a significant positive effect on digital creativity (*β* = 0.390, *p* < 0.001). Therefore, H3 was supported.

**Table 4:** Path analysis results (single-level).

Variable	Constructive deviance	Relative deprivation	Employee digital creativity
Gender	−0.077 (0.103)	0.110 (0.081)	0.081 (0.103)
Age	0.073 (0.100)	0.059 (0.075)	−0.038 (0.095)
Education level	0.089 (0.083)	−0.072 (0.065)	−0.041 (0.082)
Industry	0.005 (0.017)	−0.026 (0.014)	0.027 (0.018)
Job position	−0.010 (0.089)	−0.002 (0.065)	0.026 (0.081)
Job type	−0.055 (0.122)	−0.106 (0.088)	−0. 124 (0.114)
Tenure	−0.063 (0.091)	−0.056 (0.068)	0.027 (0.086)
Employees’ perceived involution	0.366*** (0.048)	0.399*** (0.040)	−0.371*** (0.058)
Constructive deviance			0.390*** (0.047)
Relative deprivation			−0.215** (0.067)
Residual	1.067*** (0.055)	0.631*** (0.038)	1.008*** (0.059)

*n* = 405. Standard errors are in parentheses. ***p < 0.001; **p < 0.01.

Employees’ perceived involution had a significant positive effect on relative deprivation (*β* = 0.399, *p* < 0.001). Therefore, H5 was supported. Relative deprivation had a significant negative effect on digital creativity (*β* = −0.215, *p* < 0.01). Therefore, H6 was supported.

#### 4.4.2 Testing of mediating effects

As shown in Table 5, constructive deviance played a mediating role in the relationship between Employees’ perceived involution and digital creativity [*β* = 0.140, 95% CI = (0.084, 0.197)]. Therefore, H4 was supported. Relative deprivation also played a mediating role in the relationship between Employees’ perceived involution and digital creativity [*β* = −0.086, 95% CI = (−0.142, −0.030)]. Therefore, H7 was supported.

**Table 5:** Results of the mediation effect analysis.

Path	Mediation effect	95% CI
Employees’ perceived involution → Constructive deviance → Employees’ digital creativity	0.140 (0.029)	[0.084, 0.197]
Employees’ perceived involution → Relative deprivation → Employees’ digital creativity	−0.086 (0.029)	[−0.142, −0.030]
Path	Mediation effect	95% CI

*n* = 405. Bootstrap sample size = 5000. Standard errors are in parentheses.

#### 4.4.3 Moderating effect testing

As shown in Table 6, the interaction term between Employees’ perceived involution and competitive psychological climate did not significantly affect constructive deviance [*β* = −0.052, *p* > 0.05, 95% CI = (−0.153, 0.050)]. In a highly competitive psychological climate, the effect of perceived involution on constructive deviance was weaker [*β* = 0.306, *p* < 0.001, 95% CI = (0.175, 0.437)]. In a low-competitive psychological climate, the effect of perceived involution on constructive deviance was stronger [*β* = 0.414, *p* < 0.001, 95% CI = (0.246, 0.581)]. However, the difference between these two simple slopes was not statistically significant [*β* = −0.108, *p* > 0.05, 95% CI = (−0.320, 0.105)]. Therefore, H8a was not supported, indicating that a competitive psychological climate does not moderate the relationship between Employees’ perceived involution and constructive deviance.

**Table 6:** Results of the moderating effect analysis.

Path	Moderator	Effect (SE)	95% CI
Employees’ perceived involution ×Competitive psychological climate → Constructive deviance	Competitive psychological climate	−0.052 (0.052)	[−0.153, 0.050]
	High competitive psychological climate (+1 SD)	0.306*** (0.067)	[0.175, 0.437]
	Low competitive psychological climate (−1 SD)	0.414*** (0.086)	[0.246, 0.581]
	Difference between groups	−0.108 (0.108)	[−0.320, 0.105]

*n* = 405. Bootstrap sample size = 5000. Standard errors are in parentheses. *** *p* < 0.001.

As shown in Table 7, the interaction term between employees’ perceived involution and competitive psychological climate had a significant positive effect on relative deprivation [*β* = 0.105, *p* < 0.01, 95% CI = (0.033, 0.178)]. Furthermore, under a highly competitive psychological climate, the effect of perceived involution on relative deprivation was stronger [*β* = 0.509, *p* < 0.001, 95% CI = (0.418, 0.600)]. In a climate with low levels of competition, the effect of perceived involution on relative deprivation was weaker [*β* = 0.289, *p* < 0.001, 95% CI = (0.165, 0.414)]. Moreover, the difference between these two simple slopes was statistically significant [*β* = 0.220, *p* < 0.01, 95% CI = (0.068, 0.371)]. Therefore, H8b was supported, indicating that a competitive psychological climate positively moderates the relationship between employees’ perceived involution and relative deprivation. Specifically, the stronger the competitive psychological climate is, the stronger the promoting effect of perceived involution on relative deprivation.

**Table 7:** Results of the moderating effect analysis.

Path	Moderator	Effect (SE)	95% CI
Employees’ perceived involution × Competitive psychological climate →Relative deprivation	Competitive psychological climate	0.105**(0.037)	[0.033, 0.178]
	High competitive psychological climate (+1 SD)	0.509*** (0.047)	[0.418, 0.600]
	Low competitive psychological climate (−1 SD)	0.289*** (0.064)	[0.165, 0.414]
	Difference between groups	0.220** (0.077)	[0.068, 0.371]

*n* = 405. Bootstrap sample size = 5000. Standard errors are in parentheses. **p < 0.01; ***p < 0.001.

**Figure 2:**
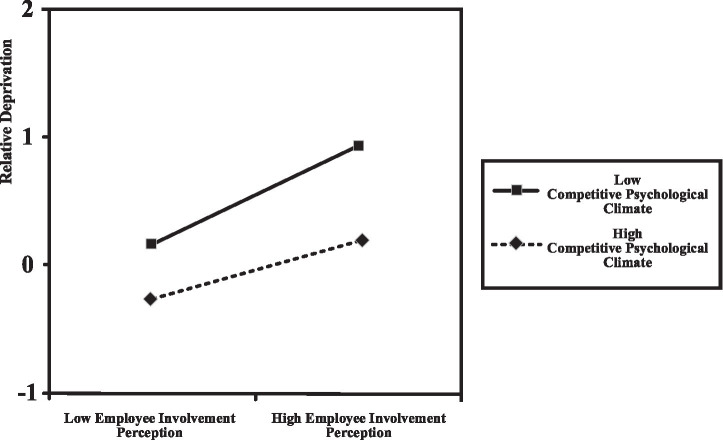
The moderating role of competitive psychological climate.

To visually illustrate the moderating effect of competitive psychological climate on the relationship between employees’ perceived involution and relative deprivation, this study plotted the relationship on the basis of the mean value plus/minus one standard deviation of the competitive psychological climate, depicting the differential effects of employees’ perceived involution on relative deprivation under varying levels of competitive psychological climate, as shown in Figure 2.

**Figure 3:**
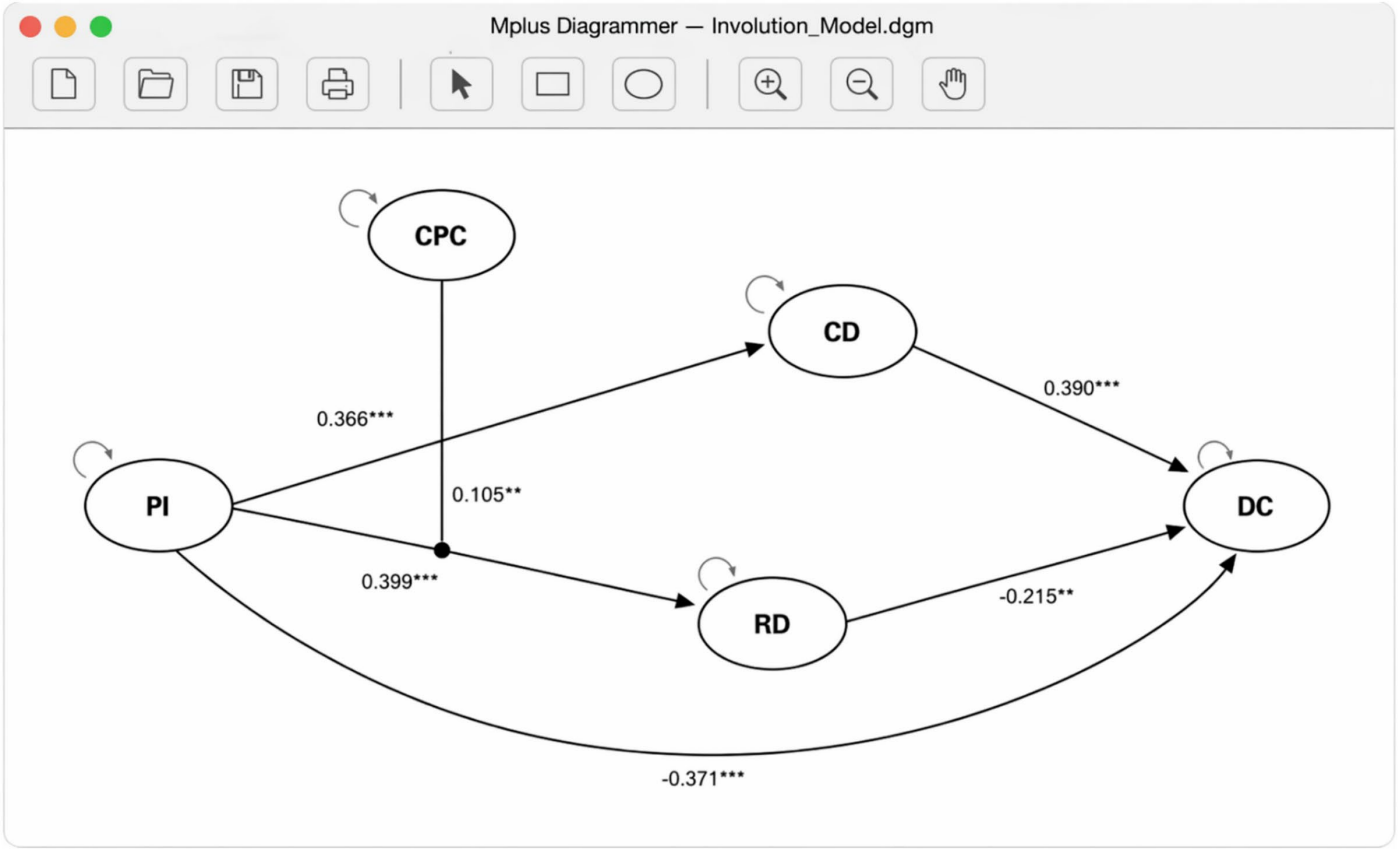
Mplus diagrammer path diagram.

The slope for the highly competitive psychological climate condition (solid line) is positive and steeper, indicating that under the highly competitive psychological climate, as employees’ perceived involution increases from low to high levels, relative deprivation increases significantly. That is, in the highly competitive psychological climate, the more employees perceive involution, the more they feel relatively deprived. Conversely, the slope for the low competitive psychological climate condition (dashed line) is positive but flatter, suggesting that under the low competitive psychological climate, relative deprivation also increases as perceived involution increases from low to high, but the magnitude of this increase is considerably smaller than that under high competition. In other words, the influence of employees’ perceived involution on relative deprivation is weaker in the weak competitive psychological climate. These results demonstrate the moderating role of the competitive psychological climate: the strong competitive psychological climate strengthens the positive relationship between employees’ perceived involution and relative deprivation, whereas the weak competitive psychological climate weakens this positive relationship. Thus, the strength of the relationship between employees’ perceived involution and relative deprivation varies significantly depending on the level of the competitive psychological climate (Figure 3).

#### 4.4.4 Test of the moderated mediation effect

**Figure 4:**
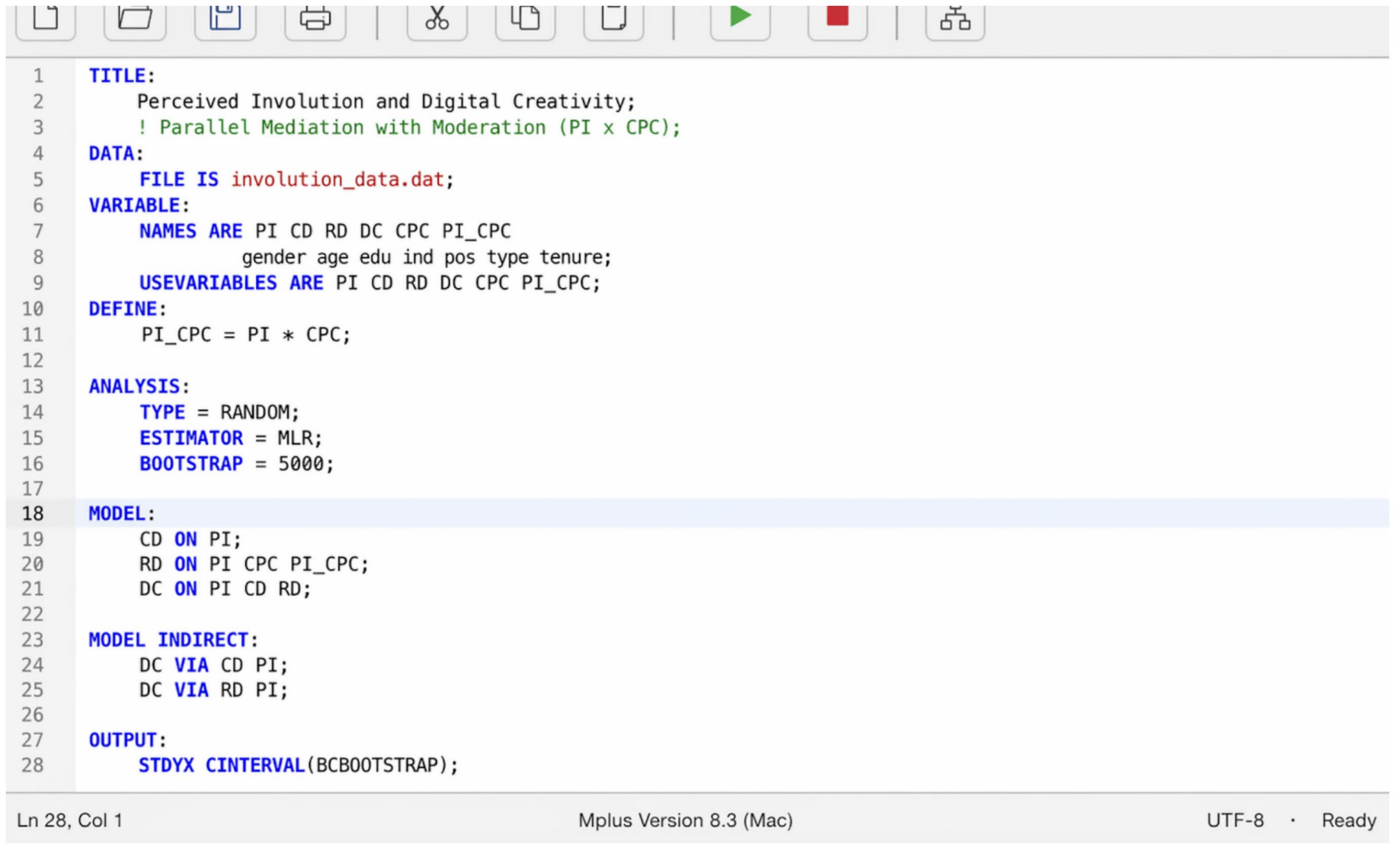
Mplus syntax editor.

As shown in Table 8, for the path “Employee’ Perceived Involutio → Constructive Deviance → Employee Digital Creativity,” the indirect relationship between Employees’ perceived involution and employee digital creativity was weaker under conditions of the competitive psychological climate [*β* = 0.066, *p* < 0.05, 95% CI = (0.015, 0.116)]. In contrast, under conditions of a low-competitiveness climate, this indirect relationship was stronger [*β* = 0.089, *p* < 0.01, 95% CI = (0.023, 0.155)]. However, the difference between the high and low climate groups was not statistically significant [*β* = −0.023, *p* > 0.05, 95% CI = (−0.073, 0.026)]. Therefore, H9a was not supported, indicating that the competitive psychological climate does not moderate the positive mediating effect of constructive deviance on the relationship between perceived involution and digital creativity (Figure 4).

**Table 8:** Results of the moderated mediation effect analysis.

Path	Moderator	Effect (SE)	95% CI
Employees’ perceived involution→ Constructive deviance → Employee digital creativity	High competitive psychological climate (+1 SD)	−0.066* (0.026)	[−0.116, −0.015]
	Low competitive psychological climate (−1 SD)	−0.089** (0.033)	[−0.155, −0.023]
	Difference between groups	0.023 (0.025)	[−0.026, 0.073]

*n* = 405. Bootstrap sample size = 5000. Standard errors are in parentheses. *p* < 0.05; **p < 0.01.

**Figure 5:**
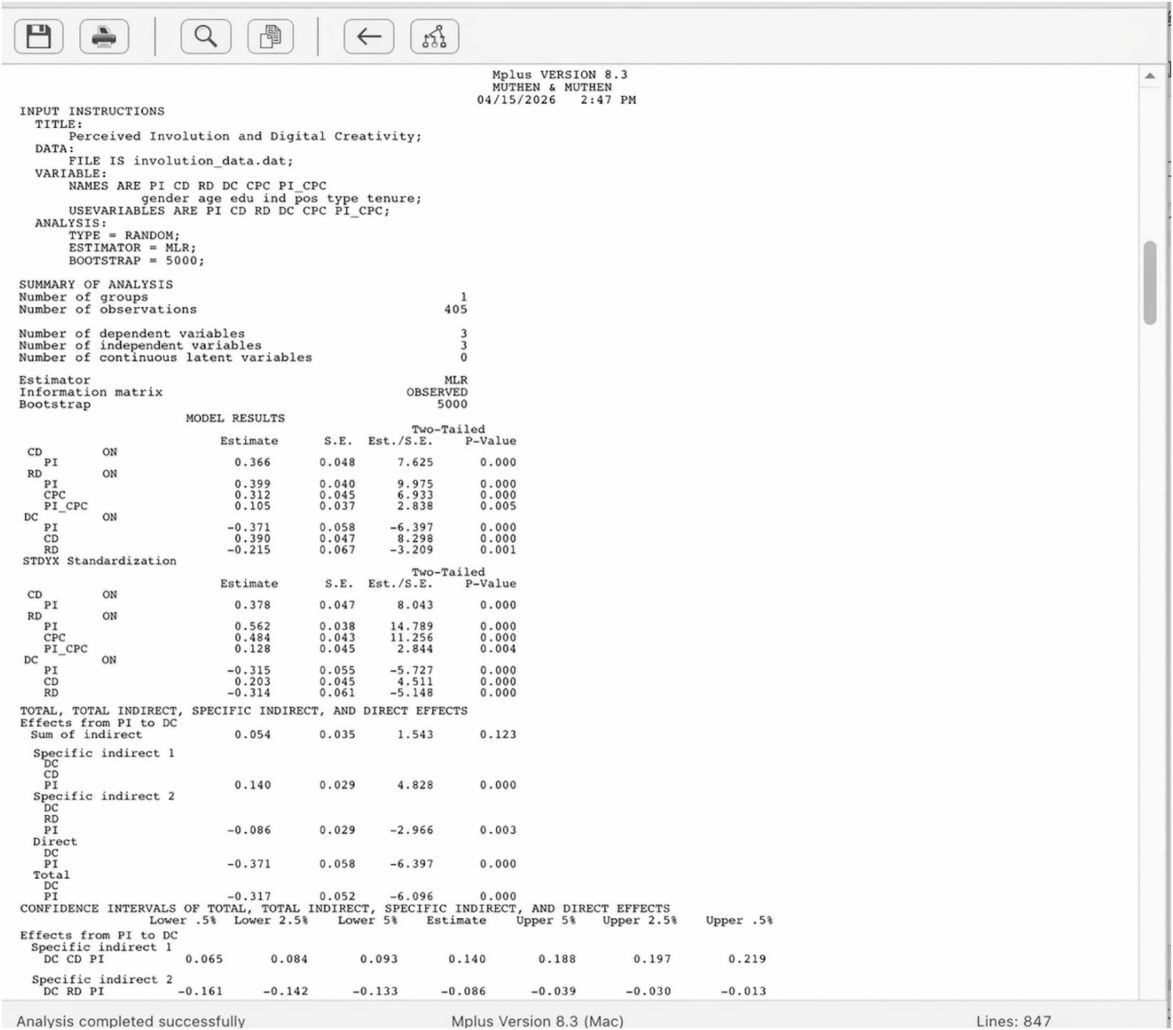
Mplus output file.

As shown in Table 9, for the path “Employees’ Perceived Involution → Relative Deprivation → Employee Digital Creativity,” the indirect relationship between employees’ perceived involution and employee digital creativity was stronger under conditions of the highly competitive psychological climate [*β* = −0.110, *p* < 0.01, 95% CI = (−0.179, −0.041)]. In contrast, under conditions of a low-competitiveness climate, this indirect relationship was weaker [*β* = −0.062, *p* < 0.05, 95% CI = (−0.112, −0.013)]. Furthermore, the difference between the high and low climate groups was statistically significant [*β* = −0.047, *p* < 0.05, 95% CI = (−0.089, −0.005)]. Therefore, H9b was supported, indicating that the competitive psychological climate positively moderates the negative mediating effect of relative deprivation on the relationship between perceived involution and digital creativity. Specifically, the stronger the competitive psychological climate is, the stronger the negative indirect effect through which Employees’ perceived involution reduces digital creativity via relative deprivation (Figure 5).

**Table 9:** Results of the moderated mediation effect analysis.

Path	Moderator	Effect (SE)	95% CI
Employees’ perceived involution → Relative deprivation → Employee digital creativity	High competitive psychological climate (+1 SD)	−0.110** (0.035)	[−0.179, −0.041]
	Low competitive psychological climate (−1 SD)	−0.062* (0.025)	[−0.112, −0.013]
	Difference between groups	−0.047* (0.022)	[−0.089, −0.005]

*n* = 405. Bootstrap sample size = 5000. Standard errors are in parentheses. *p* < 0.05; **p < 0.01.

## 5 Research conclusions and discussion

Through empirical analysis, this study validated the dual-pathway influence mechanism of employees’ perceived involution on digital creativity and its boundary conditions. Research has revealed that perceived involution significantly inhibits employees’ overall digital creativity. This influence is primarily realized through two parallel yet opposing indirect pathways: On the one hand, perceived involution can trigger a challenging response in some employees, manifested as constructive deviance such as breaking inefficient rules and integrating resources across boundaries, thereby positively promoting digital creativity. On the other hand, and more commonly, perceived involution induces feelings of relative deprivation among employees, depletes their psychological resources, and triggers defensive conservative strategies, thus significantly undermining digital creativity. Crucially, the competitive psychological climate within the organization plays an important moderating role: A high-intensity competitive psychological climate does not strengthen the path through which perceived involution enhances creativity via constructive deviance. Instead, it significantly amplifies the negative mediating chain where perceived involution exacerbates relative deprivation and subsequently inhibits digital creativity. This suggests that in a pervasively involutionary workplace, the escalation of the competitive psychological climate is more likely to exacerbate rather than ameliorate employees’ psychological states, ultimately exerting a suppressive effect on digital innovation potential.

### 5.1 Theoretical implications

1. This study breaks through the previous limitations in understanding contextual factors in creativity research. Although many studies have explored the effects of leadership styles; job design characteristics; and factors such as the digital work environment on creativity, these studies often focus on external, structural, or relatively static situational factors, severely neglecting the increasingly prominent, specific psychological context perception triggered by high-intensity competition in contemporary workplaces, especially within China’s rapidly developing digital context, namely perceived involution. Prior research failed to adequately capture employees’ subjective experience under the systematic pressure of “intensified ineffective competition, increased repetitive tasks, and compressed growth space” and its profound impact. By introducing “perceived involution,” a core construct rooted in the local cultural context reflecting individual psychological plight under high-intensity competition, into the research framework of digital creativity, this study systematically reveals, for the first time, the complex impact of this unique psychological cognition on digital innovation in a specific form. This provides a fresh perspective for understanding the psychological foundation of employee innovation in the digital economy era.
2. This study addresses the deficiency in exploring contradictory parallel pathways within the research on the mediating mechanisms of creativity. Past studies on the impact of stress or challenges on creativity have often debated a single “facilitation” or “inhibition” path or have focused only on a single mediating mechanism. In this study, self-determination theory and conservation of resources theory are creatively integrated to construct and empirically validate a parallel dual-path model containing two opposing mediating pathways. This model profoundly reveals the inherent tension in how perceived involution affects digital creativity: it can be interpreted by some employees as a challenging stressor, motivating them to seek innovative breakthroughs through constructive deviance that breaks conventions; however, more commonly, it is perceived as a hindering stressor, triggering resource loss, particularly through social comparison, generating relative deprivation, which in turn leads to defensive conservatism and creativity inhibition. This fine-grained analysis of how the same antecedent variable simultaneously affects digital creativity through distinctly different psychological and behavioral mechanisms breaks the unidimensional framework of previous research and more comprehensively depicts the complexity and contradictoriness of employee’ innovative responses in an involution context, providing a more complete theoretical model for understanding the “double-edged sword” effect of creativity generation under stressful situations.
3. This study deepens the understanding of how employees’ perception of the environment (competitive psychological climate) interacts with individual psychological perception to influence innovation, addressing the lack of refinement in moderating effects within research on perceived context. Although competitive psychological climate as a perceived environmental variable has been widely studied, its specific interaction patterns with individual perceived involution and its impact mechanisms on different innovation pathways remain unclear. Previous research might generally highlight the impact of competitive psychological climate on motivation or stress but lacks a differentiated examination of its moderating role between specific psychological cognition—perceived involution—constructive deviance and relative deprivation. This study introduces not only competitive psychological climate as a key boundary condition into the model but also its core finding that competitive psychological climate significantly strengthens the negative path through which perceived involution inhibits creativity via relative deprivation, revealing an asymmetric moderating effect. This finding indicates that in perceived high-pressure “hypercompetitive” environments, a pervasive competitive psychological climate more easily amplifies employees’ lateral comparison and perception of relative disadvantage through social information exchange, thereby exacerbating the relative deprivation pathway that undermines creativity. However, its potential promoting effect on challenging coping might be offset or suppressed by the oppressive nature of the perceived environment. This finding profoundly reveals the “unintended negative consequences” that an organizationally fostered competitive psychological climate might produce in specific perceived contexts (involution), challenging the simplistic assumption that “competition necessarily invigorates” and adds crucial and nuanced detail to the complex picture of how perceived context and individual cognition interact to influence innovation.
4. The non-significant moderating effect of competitive psychological climate on the perceived involution–constructive deviance path (H8a, H9a) is a robust finding of the present study, but the underlying mechanism cannot be directly identified from the current data. Rather than asserting a specific account, we note several non-exclusive possibilities that purpose-built future studies could adjudicate. One possibility is that constructive deviance is driven primarily by relatively stable personal dispositions (e.g., proactive personality, creative self-efficacy, psychological resilience) that were not measured in this study; if so, a climate-level moderator would have limited leverage over this path. Another possibility is that competitive climate exerts opposing forces on deviant-yet-constructive behavior—heightening the urgency to depart from ineffective routines while simultaneously raising the perceived cost of norm violation—such that the net moderating effect is attenuated. Because we did not measure dispositional moderators or separately model the motivational and sanction-related components of competitive climate, these accounts remain conjectural and are offered as directions for future research rather than as explanations of the present data.
5. This study promotes the application and development of relative deprivation theory in the field of digital innovation and strengthens its connection with local workplace phenomena. Although relative deprivation has been shown to affect general work attitudes and behaviors (e.g., satisfaction and turnover intention), its unique mechanism in the specific domain of digital creativity has not been fully explored. This study not only verifies that relative deprivation is a core mediating mechanism through which perceived involution inhibits digital creativity but also places it against the backdrop of “involution,” a phenomenon highly characteristic of the era and local context. This study contributes to the application of relative deprivation theory in the context of knowledge work and technological innovation scenarios while providing important empirical evidence from China’s transitional workplace, deepening our understanding of the formation and consequences of relative deprivation within specific cultural and social contexts.

### 5.2 Managerial implications and suggestions

1. Small and medium-sized enterprises (SMEs) must clearly recognize that the prevalent involutionary pressure among employees and an overly competitive psychological climate are critical triggers stifling creativity. Managers should start with organizational culture, and their top priority is to carefully evaluate and adjust management practices that may exacerbate cutthroat competition, particularly those overly reliant on performance ranking and last-place elimination systems. Such mechanisms tend to intensify explicit comparisons and generate a sense of relative deprivation. Enterprises should reconstruct their performance systems by reducing their reliance on short-term rankings; increasing the weight of indicators related to collaborative behaviors, long-term value, and growth potential; and striving to build an incentive ecosystem that emphasizes the joint growth of individuals and teams, encourages collaboration, and focuses on long-term contributions, thereby weakening organizational inducements for harmful competition.
2. Actively fostering a work environment with high levels of psychological safety is crucial. This means that enterprises need to provide sufficient resource support to ensure that employees can innovate without worries. Clearly, they can tolerate reasonable trial-and-error risks, viewing innovation failures as learning opportunities rather than grounds for punishment, and establish clear mechanisms for recognizing the value of innovation, allowing employees to tangibly perceive the value of breakthrough contributions. These measures can significantly reduce employees’ defensive conservatism stemming from fear of failure or concerns about effort–reward imbalance, effectively buffering the negative impact of relative deprivation on digital innovation willingness.
3. Enterprises must incorporate employee mental health support as a long-term strategy into routine management. Faced with the widespread anxiety and psychological exhaustion caused by the involution environment, enterprises should proactively provide professional stress management training and psychological counselling services and establish open, smooth, multichannel communication mechanisms for voicing concerns. These systematic initiatives can comprehensively help employees effectively cope with high-pressure environments, continuously enhancing their psychological resilience and recovery capacity, laying a solid physical and mental foundation for maintaining work passion and stimulating innovative vitality, thereby driving sustained organizational innovation.

Through the above multidimensional, systematic interventions, enterprises need to transform the organizational ecology from the vicious cycle of “internal consumption” to build a new environment characterized by benign, moderate competition, emphasizing open collaboration and value cocreation. Only in this way can employees’ precious energy and intelligence be released from futile, ineffective competition and truly focused on innovative activities that use digital technology to create substantive value, driving the sustainable development of enterprises in the digital era.

### 5.3 Limitations and future research directions

Although this study obtained findings with theoretical and practical significance, certain limitations remain. First, the study used cross-sectional data. Although collecting data at different time points mitigated common method bias, establishing strict causal relationships between variables is difficult. Although a two-wave time-lagged design was employed to collect data with a three-week interval between waves, which helps reduce common method bias (Podsakoff et al., 2003), the design remains quasi-cross-sectional in nature. The temporal separation between measurements does not fully establish strict causal ordering among the variables, as perceived involution and the mediators may fluctuate dynamically over time. Reverse causality remains a possibility, as employees who experience lower digital creativity may retrospectively attribute this to involutionary pressures. Future research should adopt multi-wave longitudinal panel designs or experience sampling methods to trace the dynamic evolution of perceived involution, mediating mechanisms, and digital creativity over extended periods, thereby strengthening causal inference. Future research should employ longitudinal tracking or experimental designs to more clearly reveal the dynamic evolution process among perceived involution, mediating variables, and digital creativity.

A related concern is that most variables in the present study, with the exception of digital creativity, were measured through employee self-reports. Although the time-lagged design and the use of supervisor-rated digital creativity partially alleviate common method variance, self-reported measures of perceived involution, relative deprivation, constructive deviance, and competitive psychological climate remain susceptible to social desirability bias and retrospective rationalization (Conway and Lance, 2010). Future studies could incorporate multi-source assessments, such as peer ratings of constructive deviance or objective indicators of competitive psychological climate derived from organizational performance records, to triangulate self-reported data and strengthen construct validity. The sample was drawn from employees in small and medium-sized manufacturing and internet enterprises in specific regions of southwestern and central China (Chongqing, Sichuan, and Shanxi). This geographic and sectoral focus may limit the generalizability of the findings. The involution phenomenon, while pervasive in China, is rooted in specific cultural and institutional conditions, including the highly competitive gaokao-driven educational pipeline, rapid but uneven digital transformation across regions, and Confucian-influenced expectations regarding diligence and social comparison (Chen Y. et al., 2024). Whether the dual-pathway mechanism identified in this study replicates in Western organizational contexts, where cultural values of individualism may alter the dynamics of social comparison and constructive deviance, remains an open question. Large enterprises with more formalized innovation management processes may also exhibit different patterns. Future research should test the model across diverse cultural settings, enterprise sizes, and industries to establish the boundary conditions of these findings. Cross-cultural comparative designs would be particularly valuable for determining whether the asymmetric moderating effect of competitive psychological climate on the relative deprivation pathway is a universal organizational phenomenon or one that is contingent on collectivist cultural values. Third, given the unexpected result that the mediating path of “constructive deviance” was not moderated by competitive psychological climate, the underlying reasons-such as whether other unmeasured perceived contextual variables or individual traits buffered/strengthened this path-require deeper qualitative or quantitative research to explore.

An additional limitation concerns the omission of individual-level dispositional and competency variables that may shape how employees respond to perceived involution. Research has consistently demonstrated that personality traits, particularly openness to experience and conscientiousness, significantly predict employee creative behavior (Sung and Choi, 2009; Anderson et al., 2014). Digital competence, defined as the ability to use digital technologies strategically and critically, represents another potentially important moderator, as employees with higher digital literacy may possess greater cognitive resources to engage in digital creativity even under involutionary pressure (van Laar et al., 2020). Proactive personality and creative self-efficacy are also likely to influence whether employees reappraise involution as a challenge or a threat (Tierney and Farmer, 2002). The present study controlled for demographic variables but did not include these individual difference factors. We acknowledge that incorporating such variables would yield a more comprehensive person–situation interaction model. This represents an important avenue for future research and has the potential to provide a more fine-grained understanding of which employees are most vulnerable to the negative effects of involution and which are most capable of converting perceived competitive pressure into creative output.

## Data Availability

The raw data supporting the conclusions of this article will be made available by the authors, without undue reservation.
